# The Gut Microbiome and Metabolites Are Altered and Interrelated in Patients With Rheumatoid Arthritis

**DOI:** 10.3389/fcimb.2021.763507

**Published:** 2022-01-25

**Authors:** Die Yu, Juping Du, Xia Pu, Liyuan Zheng, Shuaishuai Chen, Na Wang, Jun Li, Shiyong Chen, Shaobiao Pan, Bo Shen

**Affiliations:** ^1^ Department of Clinical Laboratory, Taizhou Hospital of Zhejiang Province Affiliated to Wenzhou Medical University, Taizhou, China; ^2^ Department of Rheumatology and Immunology, Taizhou Hospital of Zhejiang Province Affiliated to Wenzhou Medical University, Taizhou, China

**Keywords:** rheumatoid arthritis, autoimmune disease, gut microbiome, metabolome, biomarker, inflammation

## Abstract

The relationship among the gut microbiome, global fecal metabolites and rheumatoid arthritis (RA) has not been systematically evaluated. In this study, we performed 16S rDNA sequencing and liquid chromatography-tandem mass spectrometry (LC-MS/MS)-based nontargeted metabolomic profiling on feces of 26 untreated RA patients and 26 healthy controls. Twenty-six genera and forty-one MS2-identified metabolites were significantly altered in the RA patients. *Klebsiella*, *Escherichia*, *Eisenbergiella* and *Flavobacterium* were more abundant in the RA patients, while *Fusicatenibacter*, *Megamonas* and *Enterococcus* were more abundant in the healthy controls. Function prediction analysis demonstrated that the biosynthesis pathways of amino acids, such as L-arginine and aromatic amino acids, were depleted in the RA group. In the metabolome results, fecal metabolites including glycerophospholipids (PC(18:3(9Z,12Z,15Z)/16:1(9Z)), lysoPE 19:1, lysoPE 18:0, lysoPC(18:0/0:0)), sphingolipids (Cer(d18:0/16:0), Cer(d18:0/12:0), Cer(d18:0/14:0)), kynurenic acid, xanthurenic acid and 3-hydroxyanthranilic acid were remarkably altered between the RA patients and healthy controls. Dysregulation of pathways, such as tryptophan metabolism, alpha-linolenic acid metabolism and glycerophospholipid metabolism, may contribute to the development of RA. Additionally, we revealed that the gut microbiome and metabolites were interrelated in the RA patients, while *Escherichia* was the core genus. By depicting the overall landscape of the intestinal microbiome and metabolome in RA patients, our study could provide possible novel research directions regarding RA pathogenesis and targeted therapy.

## Introduction

Rheumatoid arthritis (RA) is a chronic, complex and systemic autoimmune disease. It is characterized by autoantibody production, synovitis, and long-standing inflammation (Scott et al., 2010). Its pathogenetic mechanism remains obscure. The gut microbiome influences the health of the host, especially with regard to gut immune homeostasis. Mounting evidence has suggested that dysbiosis of the intestinal microbiome is a vital environmental element that triggers the onset of RA, and dysregulation of the microbiome may result in abnormal immune responses (Kau et al., 2011; Taneja, 2014). It has been reported that mucosal microbes correlate with RA in animal models (van den Broek et al., 1992). In recent years, an increasing number of studies have tried to gain insight into gut microbiome interactions in RA patients. For instance, *Prevotella copri* was enriched and exhibited genomic rearrangement in new-onset untreated RA patients, and one of its 27-kDa proteins could stimulate the Th1 response in 42% of RA patients (Scher et al., 2013; Pianta et al., 2017). Some probiotics, such as *Lactobacillus casei*, significantly attenuate the expression of interferon gamma (IFN-γ), tumor necrosis factor alpha (TNF-α) and interleukin (IL)-1β to prevent joint damage (Pan et al., 2019). On the other hand, evidence indicates that microbial metabolites are crucial intermediate factors connected with the intestinal microbiome and the host. Intestinal microbes ferment food to produce numerous metabolites. Gut metabolites may pass through the mucosal barrier into the circulation to calibrate our immune system. A deficiency of beneficial bacteria and their metabolites may stimulate the inflammatory response (Velasquez-Manoff, 2015).

Combined studies of the gut microbiome and metabolome suggested promising prospects for the development of biomarkers. Unraveling the interactions between the gut microbiome and metabolome could provide new insights to discover novel targets for the treatment of various inflammatory diseases (Yang et al., 2019; Yang and Cong, 2021). Some studies of the interplay between the human gut microbiome and metabolism in RA patients have been conducted. For example, stool butyrate levels were reduced in RA patients compared to healthy controls, and supplementation with butyrate suppressed arthritis severity in an antigen-induced arthritis mouse model (Rosser et al., 2020). However, these studies mainly focused on short-chain fatty acids (SCFAs), with few studies focusing on the full spectrum of fecal metabolites. The association between the intestinal microbiome and metabolites has not been comprehensively evaluated in RA patients to date. Therefore, we performed both 16S rDNA sequencing and liquid chromatography-tandem mass spectrometry (LC-MS/MS)-based nontargeted metabolomic profiling on fecal samples of RA patients to identify potential biomarkers and microbiota-metabolite interactions to provide novel research directions for the pathogenesis of RA.

## Materials And Methods

### Recruitment of Subjects and Collection of Fecal Samples

Twenty-six RA patients were recruited from Taizhou Hospital of Zhejiang Province from July to December 2020. All patients were newly diagnosed with RA based on the RA criteria of the American College of Rheumatology. These patients did not take any disease-modifying antirheumatic drugs, biological agents or steroid drugs three months before the diagnosis. Laboratory parameters and general clinical information were obtained from our diagnosis records. In addition, 26 sex- and age-matched healthy controls (HCs) were enrolled from the medical examination center, and these HCs did not have a history of RA or abnormal inflammatory biomarkers. All patients and HCs were excluded if they (i) received any antibiotic or probiotic therapy within three months before recruitment; (ii) suffered from malignant tumors, diabetes, inflammatory bowel disease (IBD) or other autoimmune diseases; or (iii) were on an extreme diet. Furthermore, an additional 11 RA patients and 11 healthy subjects were included as the validation cohort according to the same criteria from September to October, 2021. Informed consent was obtained from all participants. The study complied with all relevant national regulations and institutional policies and was approved by the Institutional Review Board of the Ethics Committee, Taizhou Hospital of Zhejiang Province. Fecal samples were collected using sterile stool containers. Each sample was split into two tubes and stored at -80°C until subsequent processing.

### DNA Extraction and 16S rDNA Sequencing

The omics analysis was supported by LC-Bio Technology Co., Ltd, Hangzhou, Zhejiang Province, China. E.Z.N.A. ^®^Stool DNA Kit (D4015, Omega, Inc., USA) was applied to extract DNA. Then, the V3-V4 region of the 16S rRNA gene was amplified with primers 341F (5’-CCTACGGGNGGCWGCAG-3’) and 805R (5’-GACTACHVGGGTATCTAATCC-3’) (Logue et al., 2016). AMPure XT beads (Beckman Coulter Genomics, Danvers, MA, USA) and Qubit (Invitrogen, USA) were used for the purification and quantification of the polymerase chain reaction (PCR) products respectively. Finally, the Illumina NovaSeq platform was used for DNA sequencing.

### Analysis of 16S rDNA Gene Sequences

Fast length adjustment of short reads (FLASH) was applied to assign and merge paired-end reads. High-quality clean tags were obtained by FQTrim (V0.94). Operational taxonomic unit (OTU) data were then obtained based on the DADA2 algorithm (Callahan et al., 2016). SILVA and NT-16S databases were used for sequence annotation. Alpha diversity and beta diversity calculations were accomplished by QIIME2 and R (v ;3.6.1). Principal coordinate analysis (PCoA) was based on unweighted UniFrac distance, and the p-value of analysis of similarities (ANOSIM) was obtained by permutation test. Then, we conducted the Wilcoxon test and linear discriminant analysis (LDA) effect size (LEfSe) analysis to determine differential taxa. The validation cohort data were used to validate the biomarkers in LEfSe analysis. Phylogenetic investigation of communities by reconstruction of unobserved states 2 (PICRUST2) was applied to predict the function of the metagenome. Other graphs were produced using R (v 3.6.1).

### Metabolite Extraction and LC‐MS/MS Analysis

Briefly, 120 μL precooled 50% methanol buffer was added to each 50 mg fecal sample. Then the mixture was vortexed for 1 minute at room temperature and centrifuged at 4,000 g for 10 minutes (4 °C). The top 200 μL of each supernatant was transferred to 96‐well plates. The quality control samples were composed of 10 µL diluent from each sample.

An ultra-performance liquid chromatography system (SCIEX, UK) was applied to achieve chromatographic separations. Reversed-phase separation was performed on an ACQUITY UPLC T3 column (100 mm*2.1 mm, 1.8 µm, Waters, UK). The temperature was set at 35°C, and the flow rate was 0.4 mL/min. The mobile phase was composed of solvent A (water, 0.1% formic acid) and solvent B (acetonitrile, 0.1% formic acid). Gradient elution parameters were set as follows: 0~0.5 min, 5% B; 0.5~7 min, 5% to 100% B; 7~8 min, 100% B; 8~8.1 min, 100% to 5% B; 8.1~10 min, 5% B.

Metabolites were detected by high-resolution tandem mass spectrometer Triple-TOF5600plus (SCIEX, UK) in both negative and positive ion models. The ion source gas 1 was 60 PSI, the ion source gas 2 was 60 PSI, the curtain gas was 30 PSI. The source temperature was 650°C. The ion spray voltage floating of the positive and negative models were set as 5000 V and -4500 V, respectively. The time of flight (TOF) mass ranged from 60 to 1200 Da and was acquired in 150 ms. The 12 most abundant signals were chosen for the MS/MS scan, which exceeded a threshold of 100 counts/s. The total cycle took 0.56 s. Dynamic exclusion was 4 s. We conducted accuracy calibrations every twenty samples and quality control detections every ten samples.

Proteowizard MSConver was applied to transform the raw data files into mzXML format. Then, mzXML files were imported into XCMS software for peak picking and retention time correction. The Human Metabolome Database (HMDB) and online Kyoto Encyclopedia of Genes and Genomes (KEGG) were used to annotate the metabolites. The mass tolerance was set as 10 ppm. The MS/MS fragment data were validated by using an in-house metabolite fragment spectrum library. Metabolite quantification was then performed using metaX. Features were removed if observed in less than 50% of quality control samples or less than 20% of biological samples. The probabilistic quotient normalization (PQN) algorithm was applied for data normalization.

### Metabolomic Data Analysis

Multivariate analysis, Student’s *t*-test, and fold change (FC) values were used to identify differential features. The heatmap was generated by R (v 3.6.1) with the “ComplexHeatmap” package, and a volcano plot was produced with GraphPad Prism (v 8.0.1). KEGG pathway enrichment analysis was performed on differentially abundant metabolites by using R (v 3.6.1). The co-occurrence network graph was then generated with the igraph package of R (v 3.6.1). Values of p < 0.05 were considered significant, and the Benjamini-Hochberg (BH) method was used to obtain false discovery rate (FDR)-adjusted p-values (Q-values).

## Results

### Characteristics of the Study Participants

The demographic and clinical data of the RA and HC groups are described in **Table 1**. The sex (p > 0.05) and age (p > 0.05) of the two groups were matched. Body mass index (BMI) values were provided by 19 RA patients and 25 HCs. Based on the available data, no significant difference was found between the two groups (p > 0.05). Detailed clinical information of each enrolled subject are shown in **Supplementary Table 1**.

**Table 1:** Main demographic, clinical, and laboratory data of RA patients and healthy controls.

Characteristic	RA (n = 26)	Healthy controls (n = 26)	P-value
Female n (%)	16 (61.5)	16 (61.5)	0.999
Age, mean (median) years	53.3 (52.0)	52.9 (53.5)	0.917
BMI, mean (median)^a^ kg/m^2^	21.8 (21.1)	23.5 (23.9)	0.055
Disease course, mean (median) months	8.6 (2.0)	–	
TJC, mean (median)	5.9 (3.5)	–	
SJC, mean (median)	5.3 (2.5)	–	
DAS28-ESR, mean (median)	4.49 (4.16)	–	
RF positive n (%)	17 (65.4)	–	
RF titer, mean (median) IU/ml	93.9 (48.6)	–	
Anti-CCP positive n (%)	16 (61.5)	–	
ESR, mean (median) mm/h	37.8 (37.0)	–	
CRP, mean (median) mg/L	26.8 (14.2)	–	

a 19 RA patients and 25 healthy controls provided this data.

RA, rheumatoid arthritis; BMI, body mass index; TJC, tender joint count; SJC, swollen joint count; DAS28-ESR, disease activity score with 28 joint using erythrocyte sedimentation rate; RF, rheumatoid factor; Anti-CCP, anti-cyclic citrullinated peptide; ESR, erythrocyte sedimentation rate; CRP, C-reactive protein.

### Species Diversity of the Gut Microbiome

**Figure 1:**
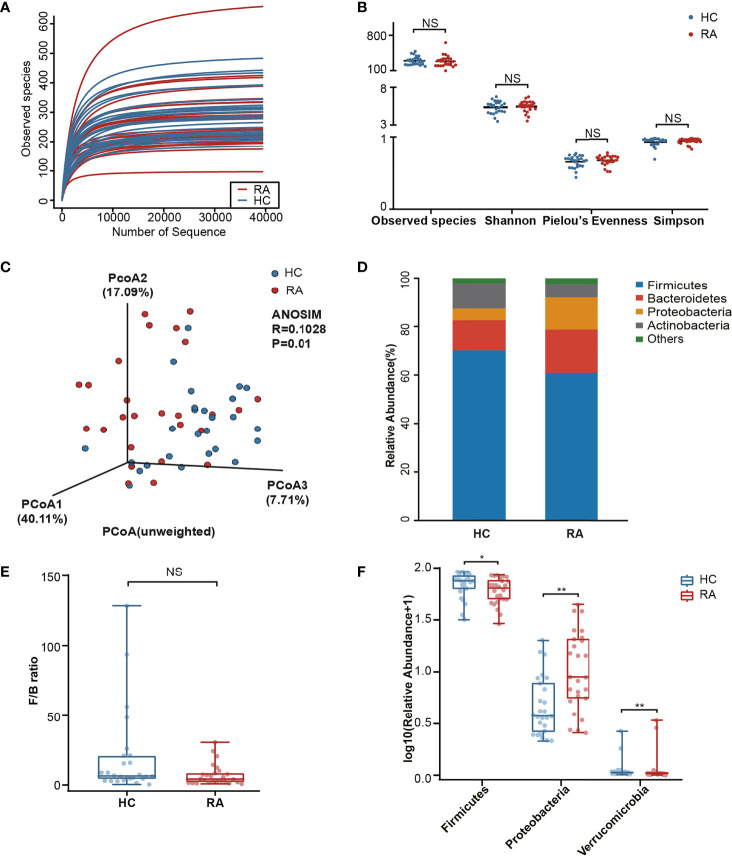
Comparison analysis of species diversity and relative abundance at the phylum level. **(A)** The curve of each sample was nearly smooth, indicating that the sequencing data was sufficient. **(B)** Alpha diversity was measured by the Observed species, Shannon, Pielou’s Evenness and Simpson index. NS, not significant. **(C)** Beta diversity was measured by principal coordinate analysis (PCoA) and analysis of similarities (ANOSIM). **(D)** The distribution plot of relative abundance at the phylum level. **(E)** The comparison of F/B ratio between the RA patients and healthy controls. NS, not significant. **(F)** The Wilcoxon test showed that three phyla were significantly altered in RA patients. P-value, *p < 0.05; **p < 0.01.

In microbiome sequencing, an average of 64,426 reads were obtained from each sample after quality filtering, and no significant difference was found between the RA patients (63,296 ± 6,711) and HCs (65,555 ± 6,340). In total, 5,804 OTUs were obtained by the DADA2 algorithm. A rarefaction curve based on the Observed species specified that the sequencing data were sufficient to detect all species in the samples (**Figure 1A**).

In our results, no significant differences in the Observed species, Shannon, Pielou’s Evenness and Simpson index were noted (**Figure 1B**) between the RA and HC groups. To further analyze the microbial composition, beta diversity was evaluated using PCoA and ANOSIM. Although the separation shown in the unweighted three-dimensional PCoA diagram was not apparent, ANOSIM revealed differences between RA patients and HCs (R = 0.1028, p < 0.05, **Figure 1C**).

### Alterations in Microbial Composition Associated With RA

The differences in the microbial composition between the two groups were evaluated at different taxonomic levels. Among the dominant phyla, Bacteroidetes and Proteobacteria were enriched in the RA patients, while Firmicutes and Actinobacteria were enriched in the HCs (**Figure 1D**). The Firmicutes/Bacteroidetes (F/B) ratio in the RA group was downregulated with no significant difference noted (p = 0.055, **Figure 1E**). The Wilcoxon test was next used to identify the significantly altered taxa. In total, 3 phyla were identified, including Firmicutes (p = 0.044), Proteobacteria (p = 0.001) and Verrucomicrobia (p = 0.006, **Figure 1F**). Among them, Verrucomicrobia was enriched in the RA group. In addition, of the 359 genera, 26 genera (p < 0.05) markedly differed in abundance between the two groups. After p-value adjustment, *Klebsiella* (Q = 0.018), *Enterococcus* (Q = 0.018) and *Eisenbergiella* (Q = 0.036) remained significant (**Supplementary Table 2**). There were 5 genera with significant differences in abundance among the top 30 richest genera. Specifically, *Escherichia* (p = 0.034) and *Klebsiella* (p < 0.001) were enriched in the RA group, while *Clostridiales_unclassified* (p = 0.034), *Megamonas* (p = 0.028) and *Fusicatenibacter* (p = 0.006) were enriched in the HC group (**Supplementary Figure 1**).

**Figure 2:**
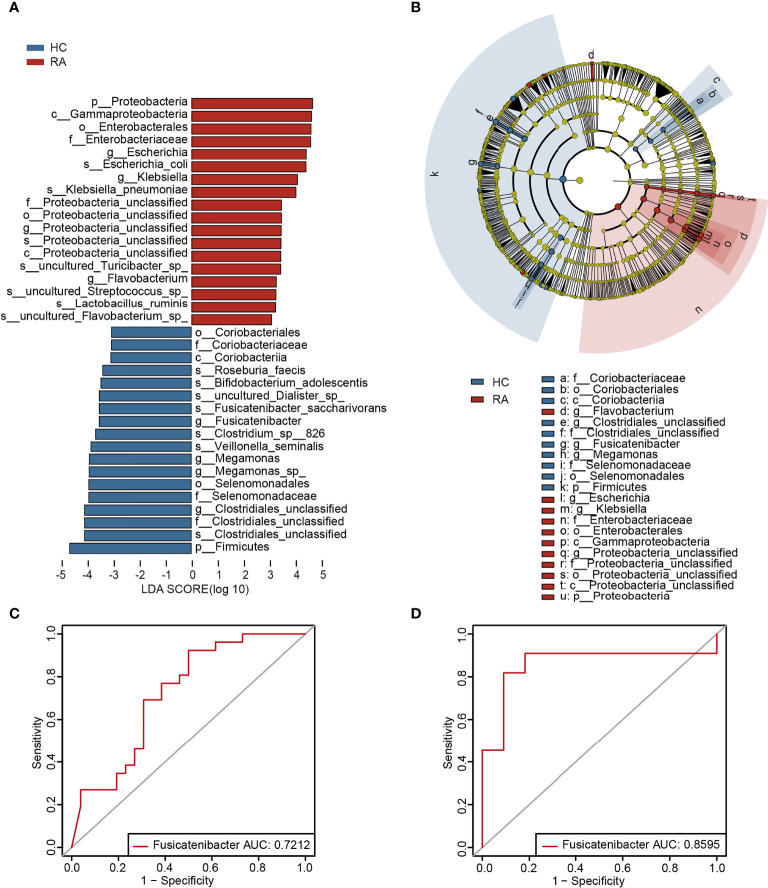
The specific altered taxa were identified by linear discriminant analysis (LDA) effect size (LEfSe) analysis. **(A)** The histogram of taxa with LDA scores more than 3 and p-value less than 0.05. **(B)** The phylogenetic tree in cladogram of the specific differential taxa. **(C)** ROC curve of *Fusicatenibacter* in test cohort. **(D)** ROC curve of *Fusicatenibacter* in validation cohort.

To further determine the specific predominant bacteria associated with RA, LEfSe was used to compare the microbial composition between the two groups. Ultimately, 36 taxa (LDA > 3, p < 0.05) were identified as significantly discriminative. Among them, *Klebsiella*, *Escherichia*, *Flavobacterium* and *Proteobacteria_unclassified* were the main taxa enriched in the RA patients, while *Fusicatenibacter*, *Megamonas, Clostridiales_unclassified* and *Coriobacteriaceae* were more abundant in the HCs (**Figures 2A, B**).

### The Potential Value of the Gut Microbiota in RA Risk Assessment

To investigate the potential of the gut microbiome in distinguishing the RA patients from the HCs, AUC values of the genera in LEfSe analysis (LDA > 3, p < 0.05) were calculated, and *Klebsiella* and *Fusicatenibacter* were ranked high (**Supplementary Table 3**). Furthermore, according to the validation cohort data (**Supplementary Table 4**), *Fusicatenibacter* was selected as a biomarker, with AUC values of 0.7212 and 0.8595 in the test cohort (**Figure 2C**) and validation cohort (**Figure 2D**), respectively.

**Figure 3:**
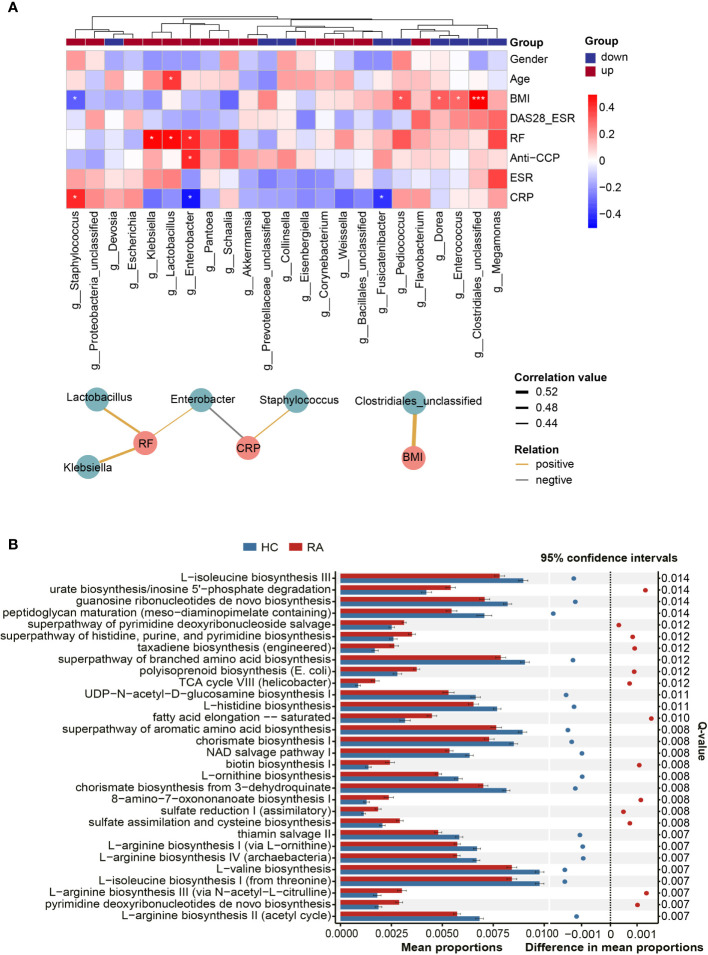
Correlations between microbes and clinical indicators and function prediction analysis. **(A)** The heatmap revealed correlations between differentially abundant genera and clinical indicators, and partial correlations (Spearman’s correlation analysis, r > 0.4, p < 0.05) were shown in co-occurrence networks. *Asaccharobacter*, *Aeromonas* and *Gelria* were excluded because they were only identified in the healthy controls. Genera were divided into two groups based on their alterations in the RA group. P-value, *p < 0.05; ***p < 0.001. **(B)** The top 30 KEGG pathways with the most significant difference in function prediction.

Based on Spearman’s correlation analysis, a heatmap was generated to show the relationship between the differentially abundant genera and clinical parameters. The main correlations (r > 0.4, p < 0.05) were further displayed in a co-occurrence network graph (**Figure 3A**). We noticed that BMI, a potential experimental confounder, had minimal effects on most of the genera except *Clostridiales_unclassified* (r = 0.56, p < 0.001). The rheumatoid factor (RF) level was positively correlated with 3 RA-enriched genera (*Klebsiella*, *Lactobacillus* and *Enterobacter*). In addition, the C-reactive protein (CRP) level was negatively correlated with *Enterobacter* and positively correlated with *Staphylococcus*.

### Microbial Function Prediction Analysis

Compared with microbial composition, microbial function seems to be more analogous in homologous environments (Gibbons, 2017). Hence, PICRUSt2 was used to infer the gene function of the microbiota. According to KEGG pathway enrichment analysis, 171 pathways were significantly altered between the RA patients and HCs (Q < 0.05, **Supplementary Table 5**). The top 30 pathways with the most significant differences are illustrated in **Figure 3B**. The results revealed that specific amino acid biosynthesis pathways were depleted in the RA group, and these amino acids included L-arginine, ornithine, aromatic amino acids, and branched amino acids. In addition, pathways such as thiamin salvage II and peptidoglycan maturation were also depleted in the RA group, while some other pathways, such as urate biosynthesis and fatty acid elongation-saturation, were enriched.

### General Overview of the Fecal Metabolome

Given that the gut microbiome could affect the immune response by producing metabolites (Krautkramer et al., 2021), LC-MS/MS-based nontargeted metabolomic profiling was performed on fecal samples of all participants. Ultimately, 1,338 features were validated through the MS2 fragment spectrum, of which 1,198 features were quantified. KEGG pathway enrichment analysis was performed, resulting in 35 significantly enriched pathways (FDR< 0.05, **Supplementary Figure 2**, **Supplementary Table 6**).

### Differentially Abundant Metabolites Between the RA and HC Groups

**Figure 4:**
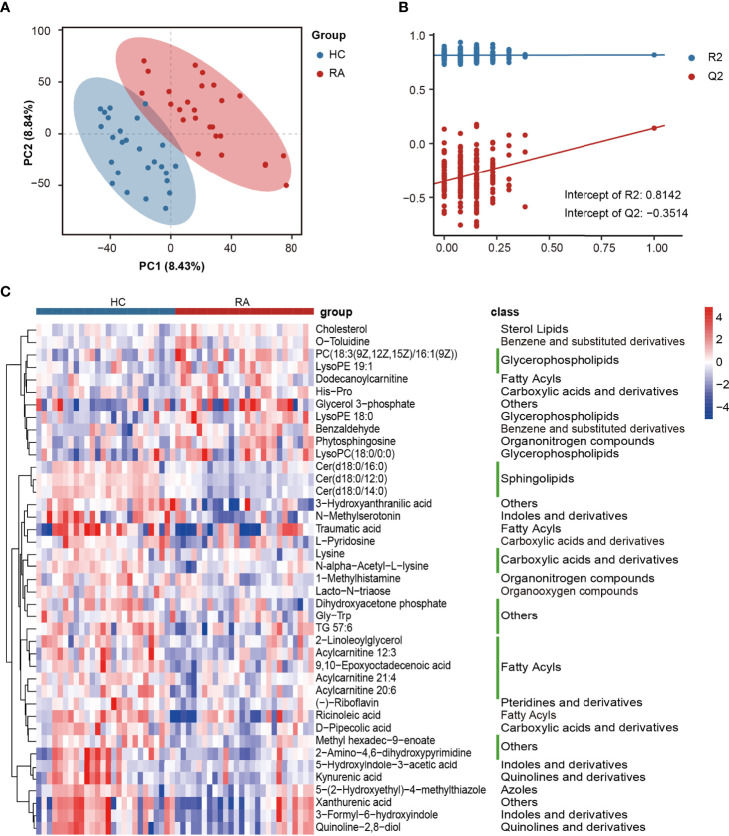
The alterations in fecal metabolites of RA patients. **(A)** PLS-DA score plot of the first two principal components. **(B)** Validation model through 200 permutation tests. **(C)** The heat map of differentially abundant metabolites based on the relative abundance.

**Figure 5:**
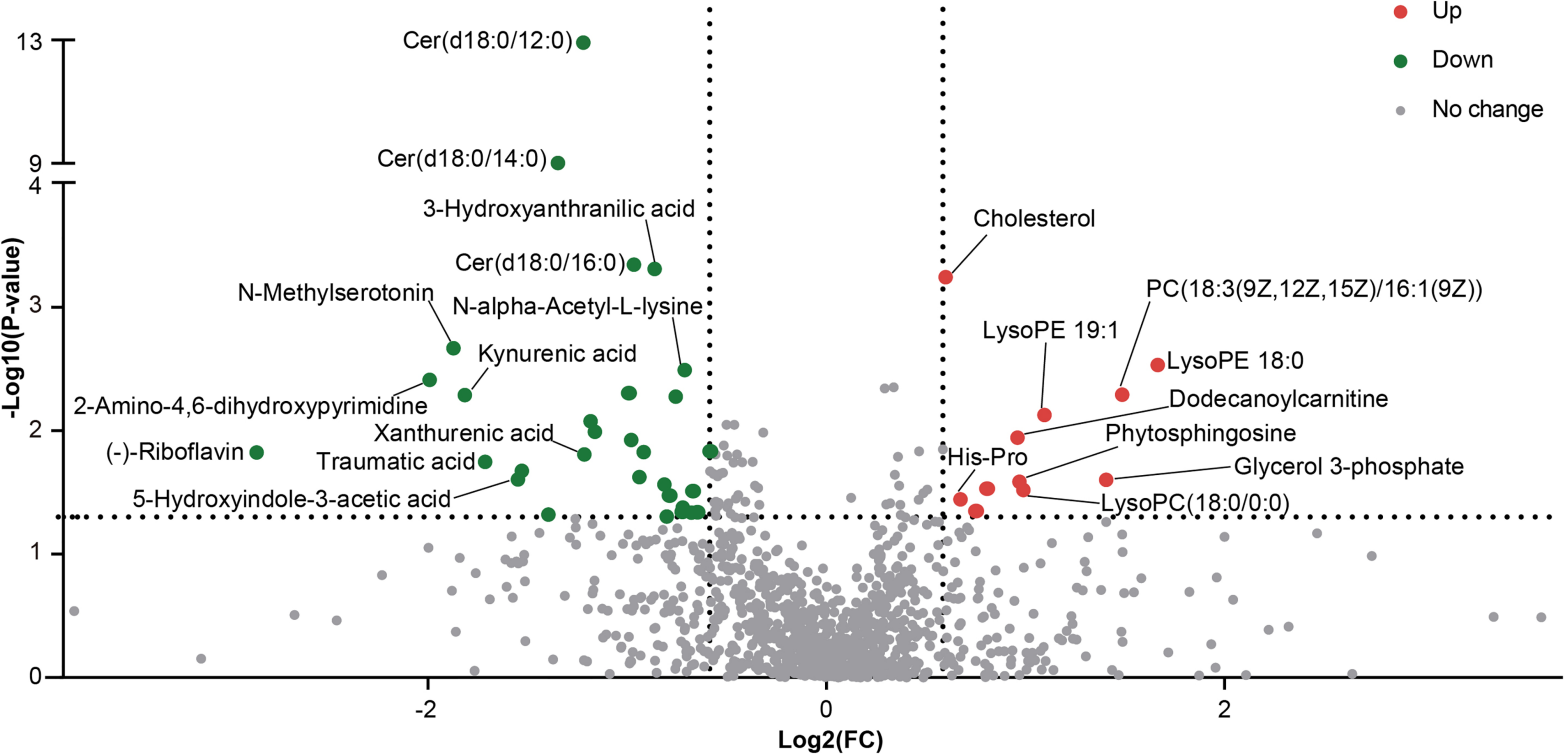
The volcano plot showed the differentially altered metabolites between the two groups.

Differentially abundant metabolites were identified based on multivariate analysis. The partial least-squares-discriminant analysis (PLS-DA) model displayed a discrimination between the RA patients and HCs according to their metabolic differences (**Figure 4A**). The permutation test indicated that the PLS-DA model was not overfitting (Intercept of Q2 = -0.3514, **Figure 4B**). Finally, 41 differentially abundant metabolites (VIP > 1, FC ≥ 1.5, p < 0.05) between the RA patients and HCs were identified. **Figures 4C** and **5** reveal the alterations of these metabolites. As a result, the RA group showed significantly high levels of glycerophospholipids (PC(18:3(9Z,12Z,15Z)/16:1(9Z)), lysoPE 19:1, lysoPE 18:0, lysoPC(18:0/0:0)), benzene and substituted derivatives (O-toluidine, benzaldehyde), cholesterol, phytosphingosine, His-Pro, glycerol 3-phosphate and dodecanoylcarnitine. In contrast, metabolites enriched in the HC group mainly including sphingolipids (Cer(d18:0/16:0), Cer(d18:0/12:0), Cer(d18:0/14:0)), fatty acyls (traumatic acid, 9,10-epoxyoctadecenoic acid, ricinoleic acid, acylcarnitine 12:3, acylcarnitine 21:4, acylcarnitine 20:6, 2-linoleoylglycerol), indoles and derivatives (N-methylserotonin, 5-hydroxyindole-3-acetic acid (5-HIAA), 3-formyl-6-hydroxyindole), kynurenic acid, xanthurenic acid, 3-hydroxyanthranilic acid (3-HAA), (-)-riboflavin and N-alpha-acetyl-L-lysine. Notably, Cer(d18:0/12:0) and Cer(d18:0/14:0) were the most significantly different molecules, and their differences remained after BH adjustment (**Supplementary Table 7**). To evaluate the possible impact of confounders (gender, age and BMI) on the differentially abundant metabolites, a correlation heat map was applied to show their relationship (**Supplementary Figure 3**). As a result, gender had remarkable associations with 2-linoleoylglycero, Gly-Trp and lacto-N-triaos, age was significantly correlated with 3-hydroxyanthranilic acid and glycerol 3-phosphate, while BMI affected these metabolites little.

**Figure 6:**
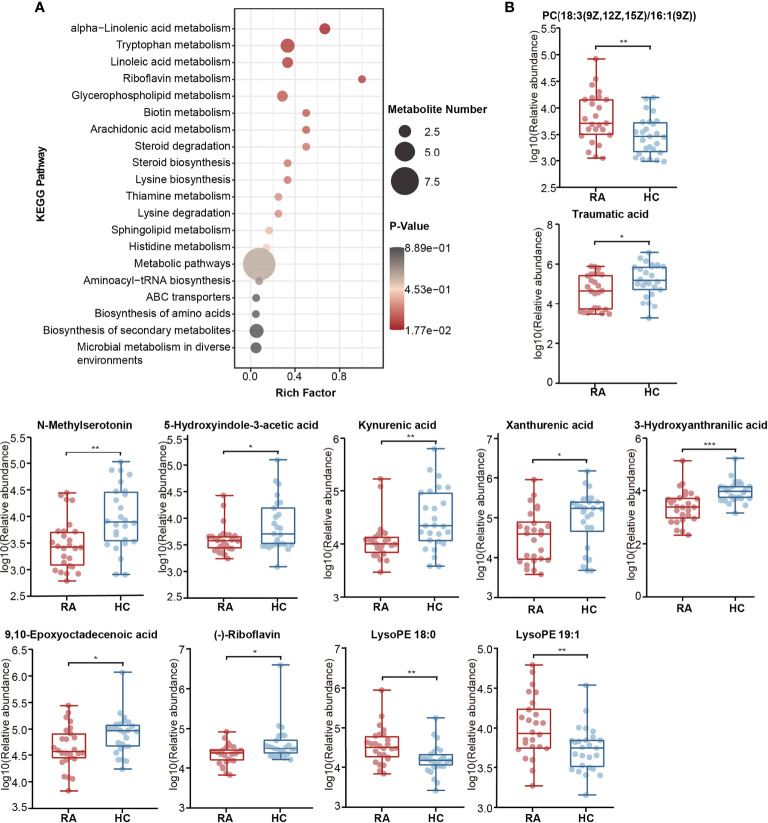
Alterations of KEGG pathway related to RA. **(A)** The KEGG pathway enrichment scatter plot displayed alterations in the intestinal metabolic processes of RA patients. **(B)** Eleven differentially abundant metabolites involved in the mainly altered pathways were further illustrated by box maps. P-value, *p < 0.05; **p < 0.01; ***p < 0.001.

KEGG pathway enrichment analysis was then performed on differentially abundant metabolites. As a result, twenty pathways, including unsaturated fatty acid (alpha-linolenic acid, linoleic acid, arachidonic acid) metabolism, tryptophan metabolism, riboflavin metabolism and glycerophospholipid metabolism were the main pathways related to RA (**Figure 6A**). The alterations of eleven differentially abundant metabolites involved in the top five pathways were further illustrated by box maps (**Figure 6B**). We noticed that the RA patients exhibited lower levels of tryptophan metabolites in feces, including N-methylserotonin, 5-HIAA, kynurenic acid, xanthurenic acid and 3-HAA.

Correspondingly, in the validation cohort, pathway enrichment analysis based on significantly altered metabolites also emphasized the importance of glycerophospholipid metabolism, linoleic acid metabolism and arachidonic acid metabolism (**Supplementary Table 8**, **Supplementary Figure 4**).

### Multiomics Analysis Revealed Microbiota-Metabolite Interactions of RA

**Figure 7:**
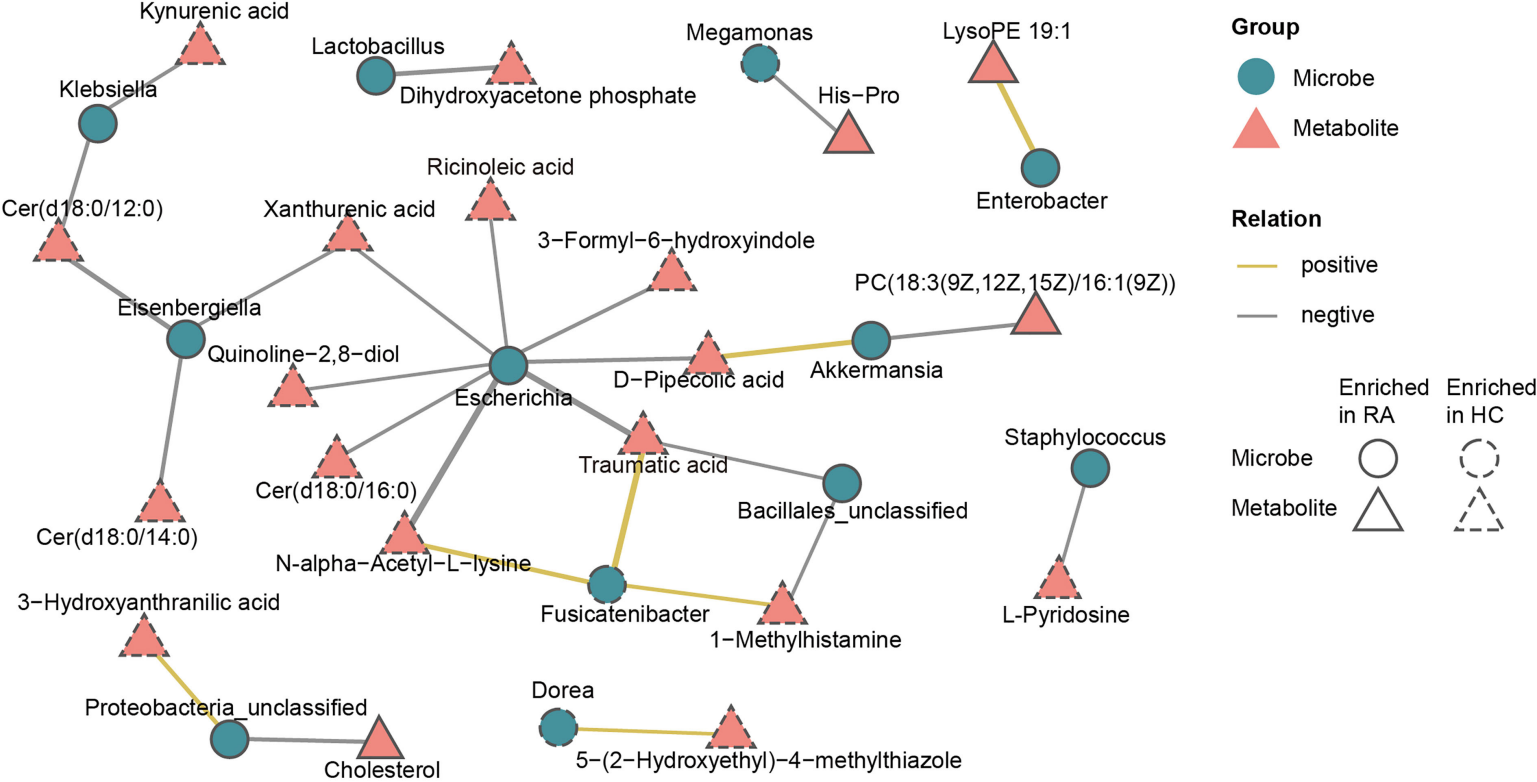
Multiomics approach revealed microbiota-metabolite interactions in RA patients. The co-occurrence network graph showed the main microbiota-metabolite correlations (Spearman’s correlation analysis, r > 0.42, P < 0.05). Microbes were labeled with circles and metabolites with triangles. Solid or dashed borders indicated that the corresponding nodes were enriched in RA patients or healthy controls, respectively. Yellow connecting lines represent positive correlations between two nodes, while gray connecting lines represent negative correlations, and thicker lines indicate greater correlation values.

To further investigate the microbiota-metabolite interactions related to RA, we evaluated the correlations between 26 genera and 41 metabolites (**Supplementary Table 9**, **Supplementary Figure 5**). Then a co-occurrence network graph was constructed to illuminate the main interplays (Spearman’s correlation analysis, r > 0.42, p < 0.05, **Figure 7**). From the graph, *Escherichia* seemed to be the core genus given that it was negatively correlated with 8 HC-enriched metabolites (ricinoleic acid, xanthurenic acid, quinoline-2,8-diol, Cer(d18:0/16:0), N-alpha-acetyl-L-lysine, traumatic acid, D-pipecolic acid, 3-formyl-6-hydroxyindole). In addition, *Eisenbergiella* was negatively correlated with HC-enriched Cer(d18:0/12:0), Cer(d18:0/14:0) and xanthurenic acid, while *Fusicatenibacter* was positively correlated with HC-enriched N-alpha-acetyl-L-lysine, traumatic acid and 1-methylhistamine. The above correlations are illustrated in **Supplementary Figure 6** using x-y plots. Other genera, such as *Klebsiella*, were also associated with certain metabolites.

## Discussion

In contrast to the metabolome of serum or urine samples, the fecal metabolome could reflect the direct interactions between dietary factors and the gut microbiome (Yang et al., 2019). Previous studies on the intestinal microbiome of RA patients lacked the attention of global fecal metabolites. Herein, we performed 16S rDNA sequencing and nontargeted metabolomic profiling to screen for neglected biomarkers and explore microbiota-metabolite interactions. Compared with the HCs, the intestinal microbiome and metabolome of the RA patients exhibited the following characteristics. (1) Microbial composition was altered. Multiple analytical approaches indicated that the main upregulated genera in the RA patients were *Klebsiella*, *Escherichia*, *Eisenbergiella* and *Flavobacterium*, and the main downregulated genera were *Fusicatenibacter*, *Megamonas* and *Enterococcus*. (2) The RA patients displayed high levels of fecal metabolites such as glycerophospholipids, benzene and substituted derivatives and cholesterol, and low levels of metabolites such as sphingolipids and tryptophan downstream metabolites. (3) The gut microbiome and metabolites were interrelated, and *Escherichia* was the core genus.

Evidence from previous studies on RA patients indicated that the alpha diversity of the intestinal microbiome was reduced or unchanged (Chu et al., 2021). Our result was consistent with the latter. Furthermore, beta diversity showed an alteration in microbial composition. We found that Proteobacteria and Verrucomicrobia were remarkably enriched in RA patients, while Firmicutes was depleted. Despite the lack of a significant difference, the RA patients had lower F/B ratios. The aberrant composition of Firmicutes and Bacteroidetes may activate proinflammatory pathways by breaking the intestinal barrier (Khan et al., 2021). We hypothesized that the F/B ratio alteration in RA patients may contribute to the inflammatory status.

In the present study, intestinal microbiota including *Klebsiella*, *Escherichia*, *Eisenbergiella*, *Flavobacterium, Lactobacillus*, and *Enterobacter* were more abundant in the RA patients. *Klebsiella* and *Escherichia* belong to the *Enterobacteriaceae* family. Consistent with our results, Li et al. also found an increased proportion of *Escherichia* in fecal samples of the RA patients (Li et al., 2021b). Furthermore, the level of anti-*Klebsiella* IgG antibodies was found increased in the serum of RA patients (Chou et al., 1998). These two kinds of bacteria can produce pathogen-associated molecular patterns (PAMPs) (Feng et al., 2019), and their lipopolysaccharide (LPS) could enhance intestinal permeability to promote inflammation (Chiang et al., 2019). In our results, *Escherichia* was negatively correlated with 8 HC-enriched fecal metabolites, including unsaturated fatty acid (ricinoleic acid and traumatic acid), D-pipecolic acid, xanthurenic acid and Cer(d18:0/16:0). *Klebsiella* was negatively correlated with HC-enriched kynurenic acid and Cer(d18:0/12:0), and exhibited a positive correlation with the serum RF. We hypothesized that the excessive expansion of *Klebsiella* and *Escherichia* contributes to a nonnegligible effect on pathogenicity in RA, which might be partly attributed to the interaction between bacteria and metabolites. *Lactobacillus-ruminis*, which has TNFα stimulatory activity (Taweechotipatr et al., 2009), was enriched in RA patients. Consistent with our results, the increase of *Lactobacillus* in the gut of chronic rheumatic disease patients has been reported previously (Salem et al., 2019). *Eisenbergiella* belongs to the Firmicutes. It was negatively correlated with HC-enriched fecal metabolites including sphingolipids (Cer(d18:0/12:0), Cer(d18:0/14:0)) and xanthurenic acid, indicating a role in promoting the development of RA. However, due to the lack of biological information, the relevant mechanism remains to be further studied.

The RA patients displayed decreases in genera such as *Enterococcus*, *Fusicatenibacter* and *Megamonas*. Enterococcus is a potential probiotic that has a wide range of inhibitory effects on pathogenic and spoilage bacteria by producing bacteriocins (Hanchi et al., 2018). The adjuvant-induced arthritis rat model demonstrated that *Enterococcus_faecium* could enhance the anti-inflammatory and antiarthritic effects of methotrexate (Rovensky et al., 2005). We hypothesized that the decrease of *Enterococcus* in RA patients may relieve the suppression of pathogenic bacteria, thus promoting a gut proinflammatory environment. *Fusicatenibacter* induces IL-10 in intestinal mucosa to exert anti-inflammatory effects (Takeshita et al., 2016). A cirrhosis fecal microbiota study specified that the abundance of *Fusicatenibacter_saccharivorans* was positively correlated with SCFA production (Jin et al., 2019), and SCFAs are beneficial to maintain the integrity of the intestinal mucosa and anti-inflammation (Davila et al., 2013; Morrison and Preston, 2016). Consistent with our study, Lee et al. also found a decrease in *Fusicatenibacter* in feces of RA patients (Lee et al., 2019). In addition, we revealed that *Fusicatenibacter* was positively correlated with HC-enriched metabolites such as traumatic acid and N-alpha-acetyl-L-lysine, and negatively correlated with serum CRP. *Megamonas*, another bacterium decreased in the RA patients, participates in the metabolism of carbohydrate fermentation into SCFAs (Feng et al., 2019). Therefore, we hypothesized that *Fusicatenibacter* and *Megamonas* might antagonize RA by affecting the abundance of SCFAs. The bacteria mentioned above might represent promising targets for RA therapy in the future and deserve further verification experiments.

Our PICRUSt2 analysis specified that the biosynthesis of amino acids, such as L-arginine, ornithine, aromatic amino acids, and branched amino acids, decreased in the RA group. Amino acids, especially branched amino acids, are precursors of SCFA synthesis in the intestinal microbiota. L-arginine has been found to alleviate inflammation in IBD patients (Baier et al., 2020). We consider that the lack of amino acid biosynthesis seems to be the main alteration of microbial function in RA patients, and this feature might partly induce an imbalance in the immune system.

In the metabolomic results, our study emphasized the crucial role of tryptophan metabolism in RA. We found that the downstream products of tryptophan metabolism, including N-methylserotonin, 5-HIAA, kynurenic acid, xanthurenic acid and 3-HAA, were depleted in the feces of the RA patients. Consistent with our findings, a study on the feces of children with enthesitis-related arthritis found downregulation of tryptophan metabolites (Stoll et al., 2016). Kang et al. also demonstrated a lower level of tryptophan metabolites in the synovial fluid of RA patients than in osteoarthritis patients (Kang et al., 2015). 5-HIAA is derived from the decomposition of serotonin and is an indole derivative. Many indole derivatives are known as ligands for aryl-hydrocarbon receptor (AhR), which can dampen immune responses by activating Treg cells (Agus et al., 2018). Rosser et al. demonstrated that microbiota-derived SCFAs could stimulate Breg cell activity and alleviate arthritis by increasing the level of 5-HIAA (Rosser et al., 2020). Kynurenic acid has an immunosuppressive function (Balog et al., 2021). It is an endogenous component of RA synovial fluid that interferes with synoviocyte proliferation *in vitro* (Parada-Turska et al., 2006). In addition, 3-HAA suppresses the LPS-induced inflammatory response in macrophages through inhibition of the nuclear factor kappa-light-chain enhancer of activated B cells (NF-κB) pathway (Lee et al., 2016). A decrease in 3-HAA was also found in the serum of RA patients (Panfili et al., 2020). Recently, studies have continually appeared suggesting that other autoimmune diseases, including systemic lupus erythematosus and multiple sclerosis, also have an obvious relationship with altered tryptophan metabolism (Brown et al., 2020). However, mechanistic studies on microbiota-derived tryptophan metabolites are lacking. Our multiomics analysis revealed the interactions between tryptophan downstream metabolites (kynurenic acid, xanthurenic acid and 3-HAA) and differential bacteria such as *Klebsiella* and *Escherichia*. The gut microbiota catabolizes tryptophan through tryptophanase and produces diverse metabolites with immune regulatory activity (Agus et al., 2018). We hypothesized that the reduced levels of fecal tryptophan metabolites may be one of the environmental risks of RA. This might be caused by complex inducements such as the redistribution of gut microbial composition and the greater gastrointestinal uptake results from the compromised gut barrier. Microbiota-derived tryptophan catabolites are expected to be developed as biomarkers for dysbiosis and provide new directions for the therapeutic target of RA.

In the present study, we also confirmed that glycero-phospholipid and unsaturated fatty acid (alpha-linolenic acid, linoleic acid and arachidonic acid) metabolism pathways were disturbed in the gut of RA patients. Under the catalysis of PLA2, glycerophospholipid is hydrolyzed to generate lysophospholipid and arachidonic acid. Subsequently, arachidonic acid is metabolized to produce inflammatory mediators such as prostaglandins and leukotrienes, which participate in the inflammatory response. Our study revealed that the fecal metabolites of the RA patients displayed significantly high levels of glycerophospholipids including PC(18:3(9Z,12Z,15Z)/16:1(9Z)), lysoPE 19:1, lysoPE 18:0 and lysoPC(18:0/0:0). Consistently, disturbance of the glycerophosphate and arachidonic acid metabolic network was also found in the serum of collagen-induced arthritis (CIA) rats (Ding et al., 2014; Li et al., 2021a). PLA2-deficient mice were not susceptible to arthritis since glycerophospholipid metabolism was restrained (Hegen et al., 2003). In contrast, as an omega-3 polyunsaturated fatty acid, alpha-linolenic acid plays a vital antithrombotic and anti-inflammatory role. Traumatic acid is a little-known beneficial compound derived from alpha-linolenic acid metabolism that has antioxidant and anti-inflammatory potential (Jablonska-Trypuc et al., 2019). In our metabolomic data, traumatic acid was significantly downregulated in the RA patients, indicating a possible antagonistic effect on RA. Furthermore, traumatic acid displayed correlations with the relative abundances of *Escherichia*, *Fusicatenibacter* and *Bacillales-unclassified*. Kindt et al. demonstrated that intestinal microbial colonization degraded dietary fiber to produce acetate, which was a precursor involved in the hepatic synthesis of unsaturated fatty acid and glycerophospholipids (Kindt et al., 2018). We suggested that the dysregulation of glycerophospholipid and unsaturated fatty acid metabolism in RA patients might be partly caused by dysbiosis of the intestinal microbiome. This mechanism may further promote the conversion of the intestinal environment from antiinflammation to proinflammation.

Other differentially abundant metabolites including cholesterol, Cer(d18:0/12:0), Cer(d18:0/14:0), (-)-riboflavin, N-alpha-acetyl-L-lysine, D-pipecolic acid and ricinoleic acid were also identified. Cholesterol is a well-known risk factor for cardiovascular disease. We observed an increase of cholesterol in the feces of RA patients, which was consistent with the results of the study based on serum (Lakatos and Harsagyi, 1988). Cer(d18:0/12:0) and Cer(d18:0/14:0) are ceramides of sphingolipids that are widely found in membranes. Bacteroidetes were the main intestinal bacteria that produce sphingolipids. Bacteroides-derived sphingolipids are beneficial for maintaining intestinal homeostasis and are negatively correlated with inflammation in IBD patients (Brown et al., 2019). Fecal metabolites of Cer(d18:0/12:0) and Cer(d18:0/14:0) was found decreased in the RA patients in our study, suggesting to be possible protective factor for RA. (-)-Riboflavin, i.e., vitamin B2, contributed strongly to maintaining gut microbiota populations. The supply of multiple vitamins is essential for the most abundant butyrate-producing Firmicutes species (Pham et al., 2021). Thus, (-)-riboflavin deficiency may lead to the depletion of SCFAs. N-alpha-acetyl-L-lysine is a lysine acetylated derivative. Lysine acetylation exists in various metabolic processes of the intestinal microbiota, including the production of SCFAs by Firmicutes. Approximately half of the lysine-acetylated derivatives in human feces were derived from Firmicutes (Zhang et al., 2020). We found that N-alpha-acetyl-L-lysine in feces of the RA patients was downregulated and significantly positively correlated with the relative abundance of *Fusicatenibacter* (a genus of Firmicutes). We hypothesized that the decrease in N-alpha-acetyl-L-lysine might be caused by the depletion of Firmicutes represented by *Fusicatenibacter*. This process may involve a decrease in SCFA production, thus affecting intestinal permeability.

Considering that diet has an essential impact on gut microbial composition, we excluded extreme diets, but we failed to control the diet composition of each participant. In addition, other confounders including gender and BMI were evaluated. Although RA is more prevalent among females than males, this study included male subjects. To eliminate gender bias, the sex ratios between the RA patients and HCs were matched. Furthermore, we checked the gender bias in correlation analyses and found a little impact on the main conclusion. BMI might influence *Clostridiales_unclassified*, which is consistent with previous studies (de La Serre et al., 2010; Kubeck et al., 2016), but has no evident impact on the genera we mainly focused on.

This study was only a cross-sectional study with a small sample size, which did not provide sufficient causality verification. According to the results, no significant difference was found in *Prevotella*, which is a research focus in RA. This finding supported the conclusion of previous studies that *Prevotella copri* increased only in the pre-or early stage of RA rather than the established RA (Vural et al., 2020). In addition, due to the low content or the unsuitable metabolite detection platform, the vital metabolite SCFAs were not identified. Expanding the duration and size of the study and performing an in-depth verification may be beneficial to further study.

Compared with single microbial data set analysis, this study presented more functional insights by introducing global metabolomic profiling, which demonstrated that the gut microbiome and metabolites were altered and interrelated in RA patients compared to HCs. The clinical verification and application of these candidate biomarkers deserve further research and development.

## Data Availability Statement

The datasets generated for this study can be found in the SRA of NCBI: https://www.ncbi.nlm.nih.gov/sra/PRJNA753264, and MetaboLights: https://www.ebi.ac.uk/metabolights/MTBLS3233.

## Ethics Statement

The studies involving human participants were reviewed and approved by Institutional Review Board of the Ethics Committee of Taizhou Hospital of Zhejiang Province. The patients/participants provided their written informed consent to participate in this study.

## Author Contributions

DY, JD, SP, and BS contributed to conception and design of the study. DY, XP, and LZ performed the experiment and statistical analysis. DY wrote the first draft of the manuscript. SSC and NW wrote sections of the manuscript. JL and SYC helped perform the analysis with constructive discussions. All authors contributed to manuscript revision and approved the submitted version.

## Funding

This work was sponsored by grants from the National Natural Science Foundation of China (No. 81672086), Zhejiang Provincial Natural Science Foundation of China (No. LY19H200001, No. LQ19H100001) and the Science and Technology Plan of Taizhou City (No. 21ywb02).

## Conflict of Interest

The authors declare that the research was conducted in the absence of any commercial or financial relationships that could be construed as a potential conflict of interest.

## Publisher’s Note

All claims expressed in this article are solely those of the authors and do not necessarily represent those of their affiliated organizations, or those of the publisher, the editors and the reviewers. Any product that may be evaluated in this article, or claim that may be made by its manufacturer, is not guaranteed or endorsed by the publisher.

## References

[B1] Agus A. Planchais J. Sokol H. (2018). Gut Microbiota Regulation of Tryptophan Metabolism in Health and Disease. Cell Host Microbe 23 (6), 716–724. doi: 10.1016/j.chom.2018.05.003 29902437

[B2] Baier J. Gansbauer M. Giessler C. Arnold H. Muske M. Schleicher U.. (2020). Arginase Impedes the Resolution of Colitis by Altering the Microbiome and Metabolome. J. Clin. Invest. 130 (11), 5703–5720. doi: 10.1172/JCI126923 32721946 PMC7598089

[B3] Balog A. Varga B. Fulop F. Lantos I. Toldi G. Vecsei L.. (2021). Kynurenic Acid Analog Attenuates the Production of Tumor Necrosis Factor-Alpha, Calgranulins (S100A 8/9 and S100A 12), and the Secretion of HNP1-3 and Stimulates the Production of Tumor Necrosis Factor-Stimulated Gene-6 in Whole Blood Cultures of Patients With Rheumatoid Arthritis. Front. Immunol. 12, 632513. doi: 10.3389/fimmu.2021.632513 33897688 PMC8062753

[B4] Brown E. M. Ke X. Hitchcock D. Jeanfavre S. Avila-Pacheco J. Nakata T.. (2019). Bacteroides-Derived Sphingolipids Are Critical for Maintaining Intestinal Homeostasis and Symbiosis. Cell Host Microbe 25 (5), 668–680 e667. doi: 10.1016/j.chom.2019.04.002 31071294 PMC6544385

[B5] Brown J. Robusto B. Morel L. (2020). Intestinal Dysbiosis and Tryptophan Metabolism in Autoimmunity. Front. Immunol. 11, 1741. doi: 10.3389/fimmu.2020.01741 32849620 PMC7417361

[B6] Callahan B. J. McMurdie P. J. Rosen M. J. Han A. W. Johnson A. J. Holmes S. P. (2016). DADA2: High-Resolution Sample Inference From Illumina Amplicon Data. Nat. Methods 13 (7), 581–583. doi: 10.1038/nmeth.3869 27214047 PMC4927377

[B7] Chiang H. I. Li J. R. Liu C. C. Liu P. Y. Chen H. H. Chen Y. M.. (2019). An Association of Gut Microbiota With Different Phenotypes in Chinese Patients With Rheumatoid Arthritis. J. Clin. Med. 8 (11), 1770. doi: 10.3390/jcm8111770 PMC6912313 31652955

[B8] Chou C. T. Uksila J. Toivanen P. (1998). Enterobacterial Antibodies in Chinese Patients With Rheumatoid Arthritis and Ankylosing Spondylitis. Clin. Exp. Rheumatol 16 (2), 161–164. 9536392

[B9] Chu X. J. Cao N. W. Zhou H. Y. Meng X. Guo B. Zhang H. Y.. (2021). The Oral and Gut Microbiome in Rheumatoid Arthritis Patients: A Systematic Review. Rheumatol. (Oxford) 60 (3), 1054–1066. doi: 10.1093/rheumatology/keaa835 33450018

[B10] Davila A. M. Blachier F. Gotteland M. Andriamihaja M. Benetti P. H. Sanz Y.. (2013). Intestinal Luminal Nitrogen Metabolism: Role of the Gut Microbiota and Consequences for the Host. Pharmacol. Res. 68 (1), 95–107. doi: 10.1016/j.phrs.2012.11.005 23183532

[B11] de La Serre C. B. Ellis C. L. Lee J. Hartman A. L. Rutledge J. C. Raybould H. E. (2010). Propensity to High-Fat Diet-Induced Obesity in Rats is Associated With Changes in the Gut Microbiota and Gut Inflammation. Am. J. Physiol. Gastrointest Liver Physiol. 299 (2), G440–G448. doi: 10.1152/ajpgi.00098.2010 20508158 PMC2928532

[B12] Ding X. Hu J. Li J. Zhang Y. Shui B. Ding Z.. (2014). Metabolomics Analysis of Collagen-Induced Arthritis in Rats and Interventional Effects of Oral Tolerance. Anal. Biochem. 458, 49–57. doi: 10.1016/j.ab.2014.04.035 24814225

[B13] Feng J. Zhao F. Sun J. Lin B. Zhao L. Liu Y.. (2019). Alterations in the Gut Microbiota and Metabolite Profiles of Thyroid Carcinoma Patients. Int. J. Cancer 144 (11), 2728–2745. doi: 10.1002/ijc.32007 30565661

[B14] Gibbons S. M. (2017). Microbial Community Ecology: Function Over Phylogeny. Nat. Ecol. Evol. 1 (1), 32. doi: 10.1038/s41559-016-0032 28812558

[B15] Hanchi H. Mottawea W. Sebei K. Hammami R. (2018). The Genus Enterococcus: Between Probiotic Potential and Safety Concerns-An Update. Front. Microbiol. 9, 1791. doi: 10.3389/fmicb.2018.01791 30123208 PMC6085487

[B16] Hegen M. Sun L. Uozumi N. Kume K. Goad M. E. Nickerson-Nutter C. L.. (2003). Cytosolic Phospholipase A2alpha-Deficient Mice are Resistant to Collagen-Induced Arthritis. J. Exp. Med. 197 (10), 1297–1302. doi: 10.1084/jem.20030016 12743172 PMC2193788

[B17] Jablonska-Trypuc A. Wydro U. Wolejko E. Butarewicz A. (2019). Toxicological Effects of Traumatic Acid and Selected Herbicides on Human Breast Cancer Cells: *In Vitro* Cytotoxicity Assessment of Analyzed Compounds. Molecules 24 (9), 1710. doi: 10.3390/molecules24091710 PMC6539929 31052542

[B18] Jin M. Kalainy S. Baskota N. Chiang D. Deehan E. C. McDougall C.. (2019). Faecal Microbiota From Patients With Cirrhosis has a Low Capacity to Ferment non-Digestible Carbohydrates Into Short-Chain Fatty Acids. Liver Int. 39 (8), 1437–1447. doi: 10.1111/liv.14106 30919578

[B19] Kang K. Y. Lee S. H. Jung S. M. Park S. H. Jung B. H. Ju J. H. (2015). Downregulation of Tryptophan-Related Metabolomic Profile in Rheumatoid Arthritis Synovial Fluid. J. Rheumatol 42 (11), 2003–2011. doi: 10.3899/jrheum.141505 26329338

[B20] Kau A. L. Ahern P. P. Griffin N. W. Goodman A. L. Gordon J. I. (2011). Human Nutrition, the Gut Microbiome and the Immune System. Nature 474 (7351), 327–336. doi: 10.1038/nature10213 21677749 PMC3298082

[B21] Khan R. Sharma A. Ravikumar R. Parekh A. Srinivasan R. George R. J.. (2021). Association Between Gut Microbial Abundance and Sight-Threatening Diabetic Retinopathy. Invest. Ophthalmol. Vis. Sci. 62 (7), 19. doi: 10.1167/iovs.62.7.19 PMC8212427 34132747

[B22] Kindt A. Liebisch G. Clavel T. Haller D. Hormannsperger G. Yoon H.. (2018). The Gut Microbiota Promotes Hepatic Fatty Acid Desaturation and Elongation in Mice. Nat. Commun. 9 (1), 3760. doi: 10.1038/s41467-018-05767-4 30218046 PMC6138742

[B23] Krautkramer K. A. Fan J. Backhed F. (2021). Gut Microbial Metabolites as Multi-Kingdom Intermediates. Nat. Rev. Microbiol. 19 (2), 77–94. doi: 10.1038/s41579-020-0438-4 32968241

[B24] Kubeck R. Bonet-Ripoll C. Hoffmann C. Walker A. Muller V. M. Schuppel V. L.. (2016). Dietary Fat and Gut Microbiota Interactions Determine Diet-Induced Obesity in Mice. Mol. Metab. 5 (12), 1162–1174. doi: 10.1016/j.molmet.2016.10.001 27900259 PMC5123202

[B25] Lakatos J. Harsagyi A. (1988). Serum Total, HDL, LDL Cholesterol, and Triglyceride Levels in Patients With Rheumatoid Arthritis. Clin. Biochem. 21 (2), 93–96. doi: 10.1016/s0009-9120(88)80094-8 3390902

[B26] Lee K. Kwak J. H. Pyo S. (2016). Inhibition of LPS-Induced Inflammatory Mediators by 3-Hydroxyanthranilic Acid in Macrophages Through Suppression of PI3K/NF-kappaB Signaling Pathways. Food Funct. 7 (7), 3073–3082. doi: 10.1039/c6fo00187d 27264984

[B27] Lee J. Y. Mannaa M. Kim Y. Kim J. Kim G. T. Seo Y. S. (2019). Comparative Analysis of Fecal Microbiota Composition Between Rheumatoid Arthritis and Osteoarthritis Patients. Genes (Basel) 10 (10), 748. doi: 10.3390/genes10100748 PMC6827100 31557878

[B28] Li Y. Lv D. Liu R. Shi Y. Wang R. Zhu Z.. (2021a). Non-Target Metabolomic Analysis Reveals the Therapeutic Effect of Saposhnikovia Divaricata Decoction on Collagen-Induced Arthritis Rats. J. Ethnopharmacol 271, 113837. doi: 10.1016/j.jep.2021.113837 33460755

[B29] Li Y. Zhang S. X. Yin X. F. Zhang M. X. Qiao J. Xin X. H.. (2021b). The Gut Microbiota and Its Relevance to Peripheral Lymphocyte Subpopulations and Cytokines in Patients With Rheumatoid Arthritis. J. Immunol. Res. 2021, 6665563. doi: 10.1155/2021/6665563 33506059 PMC7810541

[B30] Logue J. B. Stedmon C. A. Kellerman A. M. Nielsen N. J. Andersson A. F. Laudon H.. (2016). Experimental Insights Into the Importance of Aquatic Bacterial Community Composition to the Degradation of Dissolved Organic Matter. ISME J. 10 (3), 533–545. doi: 10.1038/ismej.2015.131 26296065 PMC4817675

[B31] Morrison D. J. Preston T. (2016). Formation of Short Chain Fatty Acids by the Gut Microbiota and Their Impact on Human Metabolism. Gut Microbes 7 (3), 189–200. doi: 10.1080/19490976.2015.1134082 26963409 PMC4939913

[B32] Panfili E. Gerli R. Grohmann U. Pallotta M. T. (2020). Amino Acid Metabolism in Rheumatoid Arthritis: Friend or Foe? Biomolecules 10 (9), 1280. doi: 10.3390/biom10091280 PMC7563518 32899743

[B33] Pan H. Guo R. Ju Y. Wang Q. Zhu J. Xie Y.. (2019). A Single Bacterium Restores the Microbiome Dysbiosis to Protect Bones From Destruction in a Rat Model of Rheumatoid Arthritis. Microbiome 7 (1), 107. doi: 10.1186/s40168-019-0719-1 31315667 PMC6637628

[B34] Parada-Turska J. Rzeski W. Zgrajka W. Majdan M. Kandefer-Szerszen M. Turski W. (2006). Kynurenic Acid, an Endogenous Constituent of Rheumatoid Arthritis Synovial Fluid, Inhibits Proliferation of Synoviocytes In Vitro. Rheumatol Int. 26 (5), 422–426. doi: 10.1007/s00296-005-0057-4 16220290

[B35] Pham V. T. Fehlbaum S. Seifert N. Richard N. Bruins M. J. Sybesma W.. (2021). Effects of Colon-Targeted Vitamins on the Composition and Metabolic Activity of the Human Gut Microbiome- a Pilot Study. Gut Microbes 13 (1), 1–20. doi: 10.1080/19490976.2021.1875774 PMC7899684 33615992

[B36] Pianta A. Arvikar S. Strle K. Drouin E. E. Wang Q. Costello C. E.. (2017). Evidence of the Immune Relevance of Prevotella Copri, a Gut Microbe, in Patients With Rheumatoid Arthritis. Arthritis Rheumatol 69 (5), 964–975. doi: 10.1002/art.40003 27863183 PMC5406252

[B37] Rosser E. C. Piper C. J. M. Matei D. E. Blair P. A. Rendeiro A. F. Orford M.. (2020). Microbiota-Derived Metabolites Suppress Arthritis by Amplifying Aryl-Hydrocarbon Receptor Activation in Regulatory B Cells. Cell Metab. 31 (4), 837–851 e810. doi: 10.1016/j.cmet.2020.03.003 32213346 PMC7156916

[B38] Rovensky J. Svik K. Matha V. Istok R. Kamarad V. Ebringer L.. (2005). Combination Treatment of Rat Adjuvant-Induced Arthritis With Methotrexate, Probiotic Bacteria Enterococcus Faecium, and Selenium. Ann. N Y Acad. Sci. 1051, 570–581. doi: 10.1196/annals.1361.101 16126997

[B39] Salem F. Kindt N. Marchesi J. R. Netter P. Lopez A. Kokten T.. (2019). Gut Microbiome in Chronic Rheumatic and Inflammatory Bowel Diseases: Similarities and Differences. U Eur. Gastroenterol. J. 7 (8), 1008–1032. doi: 10.1177/2050640619867555 PMC6794689 31662859

[B40] Scher J. U. Sczesnak A. Longman R. S. Segata N. Ubeda C. Bielski C.. (2013). Expansion of Intestinal Prevotella Copri Correlates With Enhanced Susceptibility to Arthritis. Elife 2, e01202. doi: 10.7554/eLife.01202 24192039 PMC3816614

[B41] Scott D. L. Wolfe F. Huizinga T. W. (2010). Rheumatoid Arthritis. Lancet 376 (9746), 1094–1108. doi: 10.1016/S0140-6736(10)60826-4 20870100

[B42] Stoll M. L. Kumar R. Lefkowitz E. J. Cron R. Q. Morrow C. D. Barnes S. (2016). Fecal Metabolomics in Pediatric Spondyloarthritis Implicate Decreased Metabolic Diversity and Altered Tryptophan Metabolism as Pathogenic Factors. Genes Immun. 17 (7), 400–405. doi: 10.1038/gene.2016.38 27786174 PMC5133160

[B43] Takeshita K. Mizuno S. Mikami Y. Sujino T. Saigusa K. Matsuoka K.. (2016). A Single Species of Clostridium Subcluster XIVa Decreased in Ulcerative Colitis Patients. Inflammation Bowel Dis. 22 (12), 2802–2810. doi: 10.1097/MIB.0000000000000972 27824645

[B44] Taneja V. (2014). Arthritis Susceptibility and the Gut Microbiome. FEBS Lett. 588 (22), 4244–4249. doi: 10.1016/j.febslet.2014.05.034 24873878 PMC4246018

[B45] Taweechotipatr M. Iyer C. Spinler J. K. Versalovic J. Tumwasorn S. (2009). Lactobacillus Saerimneri and Lactobacillus Ruminis: Novel Human-Derived Probiotic Strains With Immunomodulatory Activities. FEMS Microbiol. Lett. 293 (1), 65–72. doi: 10.1111/j.1574-6968.2009.01506.x 19222575 PMC4105522

[B46] van den Broek M. F. van Bruggen M. C. Koopman J. P. Hazenberg M. P. van den Berg W. B. (1992). Gut Flora Induces and Maintains Resistance Against Streptococcal Cell Wall-Induced Arthritis in F344 Rats. Clin. Exp. Immunol. 88 (2), 313–317. doi: 10.1111/j.1365-2249.1992.tb03079.x 1572097 PMC1554307

[B47] Velasquez-Manoff M. (2015). Gut Microbiome: The Peacekeepers. Nature 518 (7540), S3–11. doi: 10.1038/518S3a 25715278

[B48] Vural M. Gilbert B. Ustun I. Caglar S. Finckh A. (2020). Mini-Review: Human Microbiome and Rheumatic Diseases. Front. Cell Infect. Microbiol. 10, 491160. doi: 10.3389/fcimb.2020.491160 33304855 PMC7693548

[B49] Yang W. Cong Y. (2021). Gut Microbiota-Derived Metabolites in the Regulation of Host Immune Responses and Immune-Related Inflammatory Diseases. Cell Mol. Immunol. 18 (4), 866–877. doi: 10.1038/s41423-021-00661-4 33707689 PMC8115644

[B50] Yang Y. Misra B. B. Liang L. Bi D. Weng W. Wu W.. (2019). Integrated Microbiome and Metabolome Analysis Reveals a Novel Interplay Between Commensal Bacteria and Metabolites in Colorectal Cancer. Theranostics 9 (14), 4101–4114. doi: 10.7150/thno.35186 31281534 PMC6592169

[B51] Zhang X. Ning Z. Mayne J. Yang Y. Deeke S. A. Walker K.. (2020). Widespread Protein Lysine Acetylation in Gut Microbiome and its Alterations in Patients With Crohn's Disease. Nat. Commun. 11 (1), 4120. doi: 10.1038/s41467-020-17916-9 32807798 PMC7431864

