# Supplementary material for: The Gut Microbiome and Metabolites Are Altered and Interrelated in Patients With Rheumatoid Arthritis
Source: Front Cell Infect Microbiol. 2022 Jan 25;11:763507. doi: 10.3389/fcimb.2021.763507 (PMC8821809; doi:10.3389/fcimb.2021.763507)
Supplement: Supplementary file 1 [file DataSheet_1.pdf]

Supplementary Material

1. Supplementary Figures and Tables

1.1. Supplementary Figures

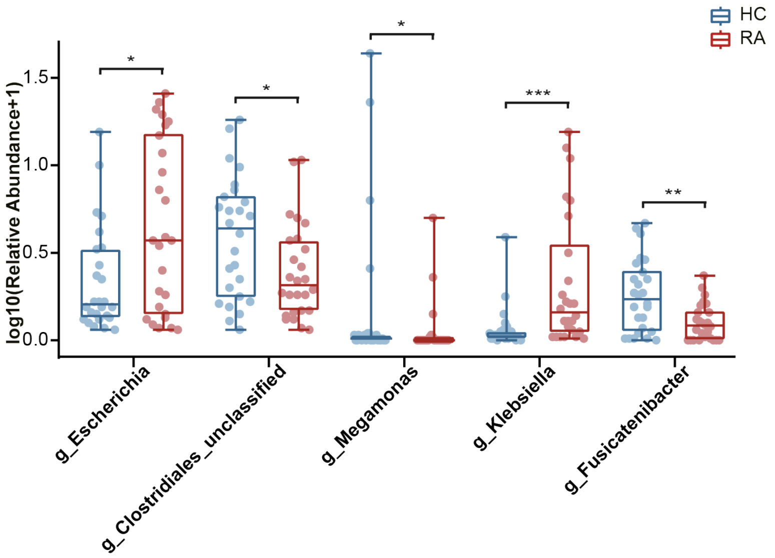

Supplementary Figure 1 The Wilcoxon test showed that 5 genera were significantly altered in RA patients among the top 30 richest genera. P-value: *p<0.05; **p<0.01 ***p<0.001

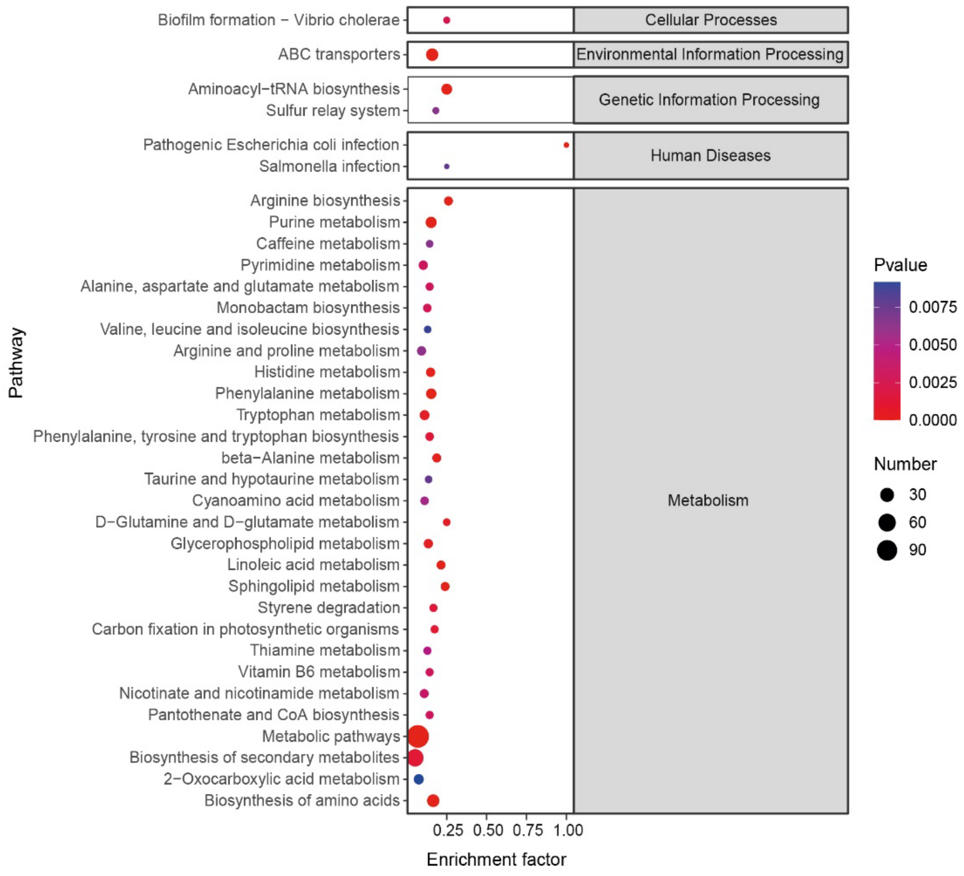

Supplementary Figure 2 The KEGG pathway enrichment analysis of MS2 metabolites. 35 significantly enriched pathways were obtained (FDR < 0.05).

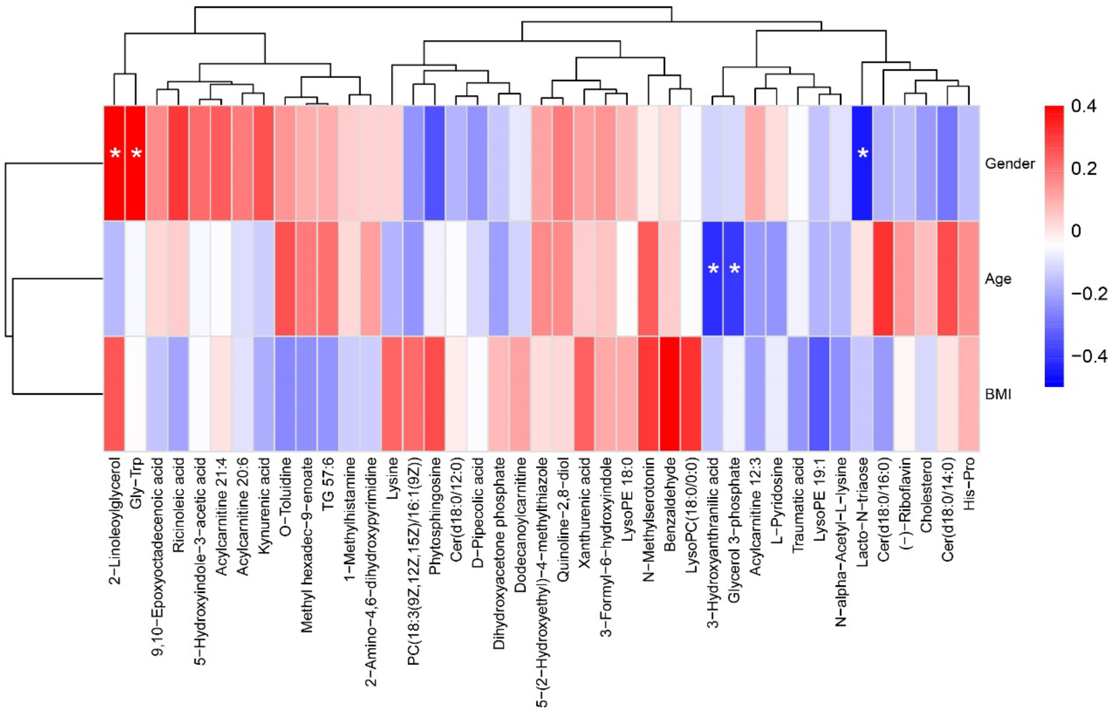

Supplementary Figure 3 The heatmap revealed the correlations between possible confounders (gender, age and BMI) and the significantly altered metabolites (Spearman's correlation analysis). P-value, *p<0.05.

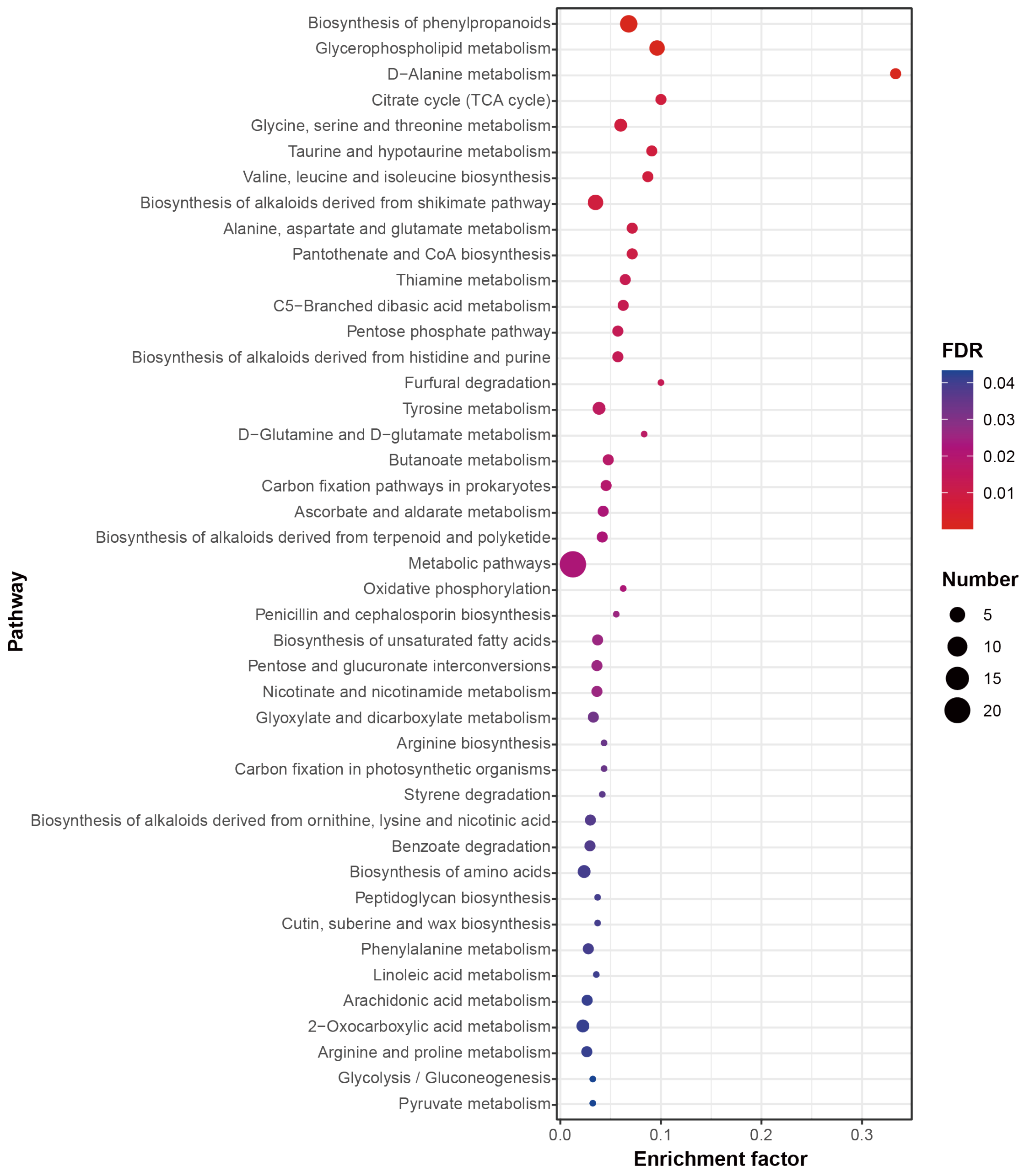

Supplementary Figure 4 The KEGG pathway enrichment analysis based on the significantly altered metabolites selected in validation cohort data.

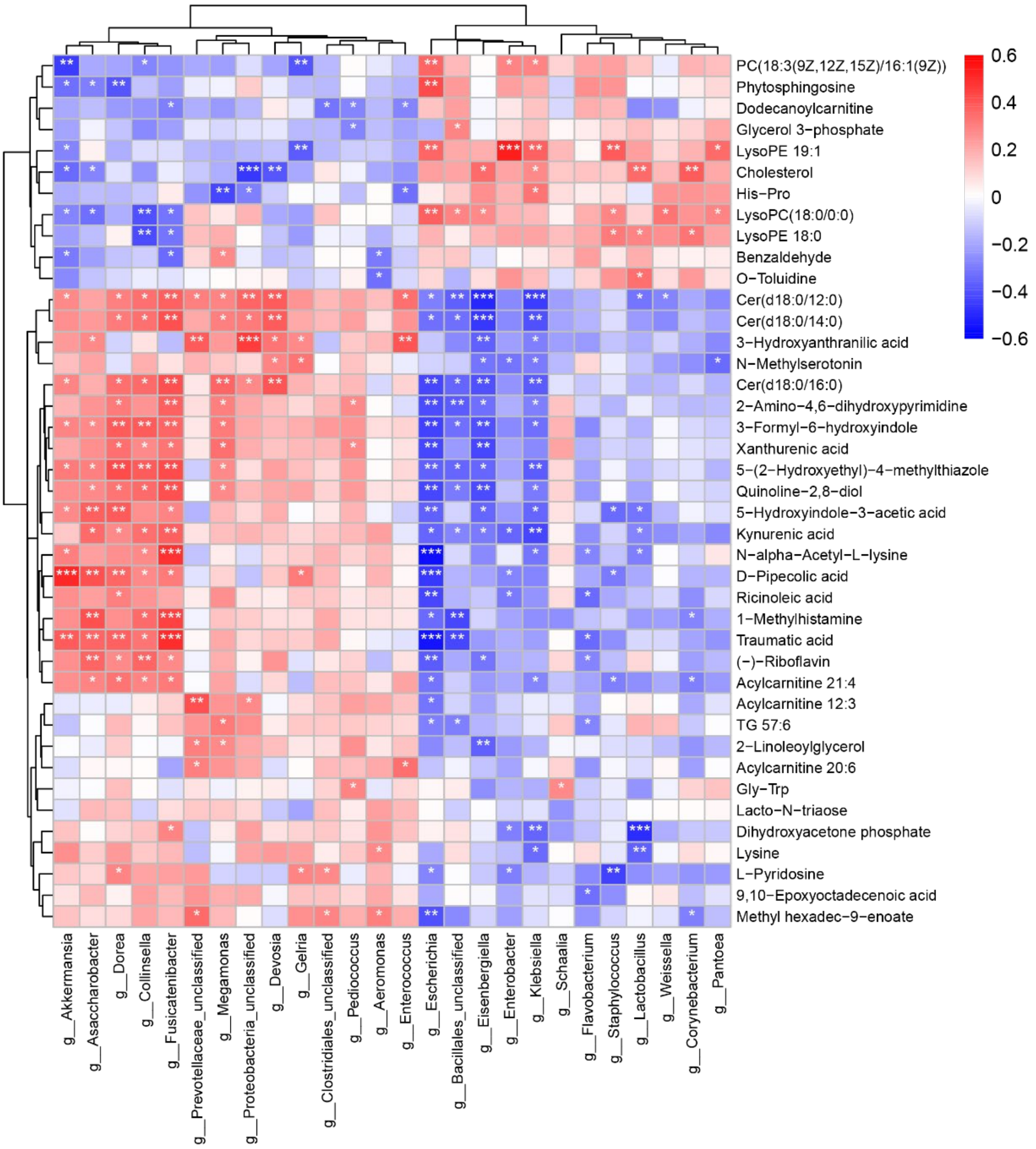

Supplementary Figure 5 Heatmap was conducted to demonstrate the correlations between 26 differentially abundant genera and 41 differentially abundant metabolites (Spearman's correlation analysis). P-value, *p<0.05; **p<0.01; ***p<0.001

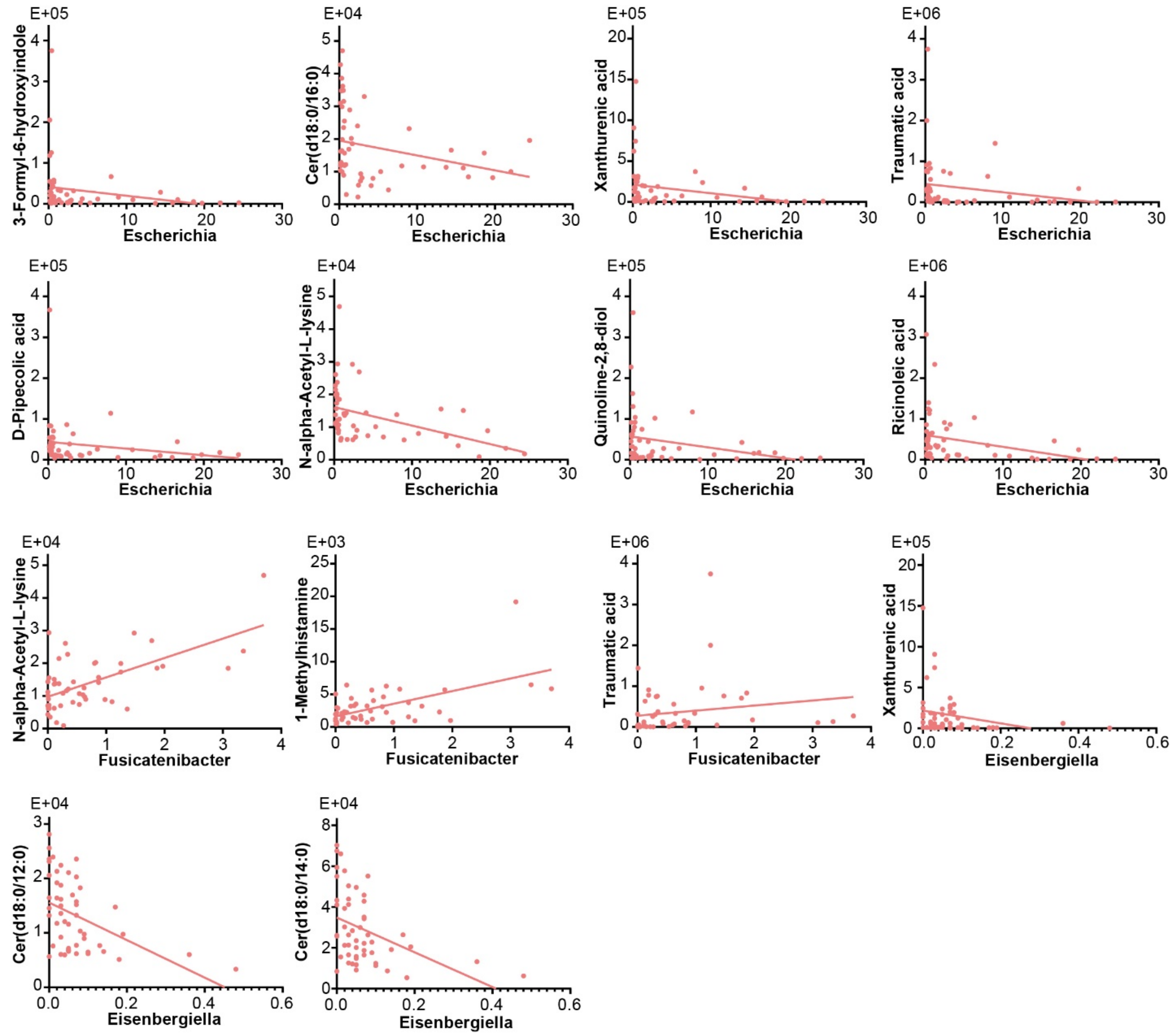

Supplementary Figure 6 The x-y plots between the predominant bacteria (*Escherichia*, *Eisenbergiella* and *Fusicatenibacter*) and their relevant significantly altered metabolites.

1.2. Supplementary Tables

Supplementary Table 1. Demographic and clinical characteristics of each enrolled subject.

Supplementary Table 2. 26 differentially abundant genera were obtained by the Wilcoxon test.

Supplementary Table 3. AUC-values of candidate genera (LEfSe analysis, LDA>3, P-value<0.05).

Supplementary Table 4. Raw data and AUC-values of *Klebsiella* and *Fusicatenibacter* in validation cohort.

Supplementary Table 5. 171 pathways were found significantly altered between the RA and HC groups.

Supplementary Table 6. 35 pathways were significantly enriched based on MS2 metabolites.

Supplementary Table 7. The classification and alteration of differentially abundant metabolites.

Supplementary Table 8. KEGG pathway enrichment results based on differentially abundant metabolites selected in the validation cohort.

Supplementary Table 9. Spearman's correlation (rho) values between differential metabolites and differential microbes.
